# The difference in the thermal conductivity of nanofluids measured by different methods and its rationalization

**DOI:** 10.3762/bjnano.7.194

**Published:** 2016-12-20

**Authors:** Aparna Zagabathuni, Sudipto Ghosh, Shyamal Kumar Pabi

**Affiliations:** 1 Department of Metallurgical and Materials Engineering, Indian Institute of Technology Kharagpur, West Bengal-721302, India

**Keywords:** Brownian movement, collision-mediated heat transfer model, laser flash method, nanofluids, thermal conductivity, transient hot-wire method

## Abstract

A suspension of particles below 100 nm in size, usually termed as nanofluid, often shows a notable enhancement in thermal conductivity, when measured by the transient hot-wire method. In contrast, when the conductivity of the same nanofluid is measured by the laser flash method, the enhancement reported is about one order of magnitude lower. This difference has been quantitatively resolved for the first time on the basis of the collision-mediated heat transfer model for nanofluids proposed earlier by our research group. Based on the continuum simulation coupled with stochastic analysis, the present theoretical prediction agrees well with the experimental observations from different measuring methods reported in the literature, and fully accounts for the different results from the two measuring methods mentioned above. This analysis also gives an indication that the nanofluids are unlikely to be effective for heat transfer in microchannels.

## Introduction

In 1995, Choi et al. [1] dispersed copper nanoparticles in water, and termed the suspension as nanofluid. They observed a large increase in the thermal conductivity of this nanofluid compared to water when measured by the transient hot-wire method (THWM). Subsequently, the thermal conductivity of nanofluids has been extensively investigated by THWM with the prospect to use them for enhanced heat-transfer applications [2–8]. However, the cause of this enhanced thermal conductivity is still under debate and, to date, many mechanisms have been proposed [9–16]. Researchers [17–22] have considered various mechanisms and concluded that all the proposed mechanisms are not adequate to predict the order of magnitude of enhancement in thermal conductivity observed experimentally. Recently Ghosh et al. [16] followed by Karthik et al. [23] proposed a model that took into consideration a crucial event, which was previously ignored or overlooked, i.e., the thermal exchange between the nanoparticles and the heat source. Incorporating the thermal exchange during these collisions, Karthik et al. [23] predicted enhancements in thermal conductivity that were in good agreement with the experimentally observed values.

Steady-state and transient methods can be used to quantify the thermal conductivity of nanofluids. The steady-state methods are not adequate, because by the time the system reaches a steady state, heat transfer through convection and heat transfer through radiation set in, which results in an inaccurate result. The transient methods, on the other hand, are designed to minimize the effects of radiation and convection by reducing the heating time and minimizing the contact area between the heat source and the liquid in a well-insulated system. The most common methods for measuring the thermal conductivity of nanofluids are the transient hot-wire method (THWM), the transient plane-source method, the laser flash method (LFM), the 3ω method, and the thermal oscillation method.

One unexplained characteristic of heat transfer in nanofluids is the big difference in the values of the thermal conductivity obtained by the laser flash method (LFM) and the transient hot-wire method (THWM). Buonomo et al. [24] have measured the thermal conductivity of water-based Al_2_O_3_ nanofluids using the LFM. They observed that at room temperature the enhancement in thermal conductivity for 4 vol % of Al_2_O_3_-nanoparticle loading was around 4.95%, whereas Beck et al. [4] obtained 16.5% enhancement using the transient hot-wire method for the same Al_2_O_3_ nanoparticle loading and particle size. Lee et al. [25] have investigated the thermal conductivity of Al_2_O_3_, SiC, Ni, ZnO and multiwalled carbon nanotubes (MWCNTs) in liquid gallium using LFM. They reported that the thermal conductivity measured by LFM was not accurate because of the uncertainty in the specific heat of the nanofluid. Using LFM, Zeng et al. [3] have reported 38.7% enhancement for 1.0 vol % loading of MoS_2_ nanoparticles in oil. They recognized that the thermal conductivity enhancement diminishes when the temperature is close to the flash point of the base oil. Based on several reports it was found that the values of thermal conductivity enhancements of nanofluids obtained by means of LFM are significantly lower than those obtained by THWM. To address the difference in the measured thermal conductivity enhancement reported in the literature an attempt has been made to explore other possible factors that might influence the observed enhancement [26]. The results manifest for the first time the profound influence of container type, nanoparticle type, base fluid and temperature on the measured thermal conductivity enhancement. It was found that these factors become almost insignificant, if the dispersed material was ceramic (typically Al_2_O_3_). In this paper we put forward a quantitative analysis based on the collision-mediated heat transfer model for water-based Al_2_O_3_ nanofluids, which can account for the difference in the thermal conductivity values of nanofluids obtained by LFM and THWM.

## Collision-Mediated Model

**Figure 1:**
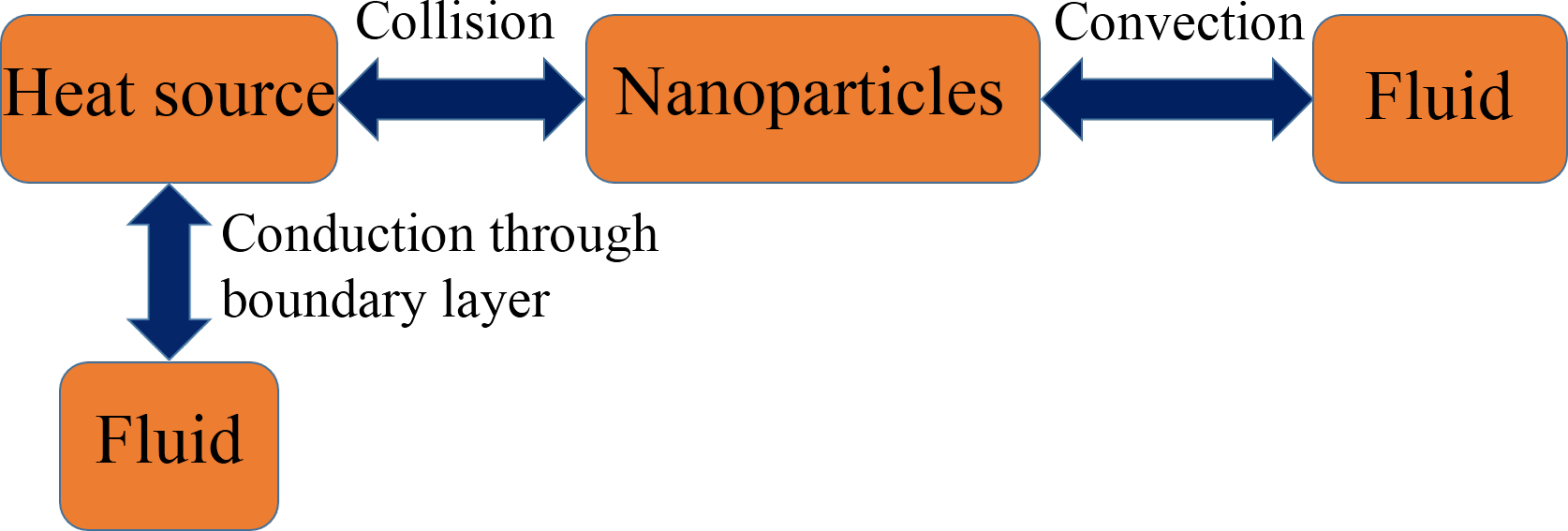
Schematic representation of collision mediated heat transfer mechanism.

Ghosh et al. [16] proposed a new mechanism for the enhancement of thermal conductivity in nanofluids. According to them, nanoparticles within nanofluids undergo Brownian motion and frequently collide with the heat source. During these collisions, rapid heat exchange occurs between the nanoparticles and heat source within few picoseconds, which almost instantaneously raises or drops the temperature of the nanoparticles. Subsequent movement of the nanoparticles in the fluid is accompanied by convective heat exchange between nanoparticles and the adjacent fluid. Figure 1 schematically illustrates this process. In addition to the conventional mechanism of heat transfer between heat source and fluid through the thermal boundary layer, the additional mechanism via nanoparticles substantially contributes to the heat flux near the heat source.

Using a stochastic Brownian model Karthik et al. [23] computed the evolution of phase space and, consequently, the frequency of collision of nanoparticles with a heat-source wall (having unit area) for a nanofluid having a given volume fraction and size distribution of nanoparticles. Using classical molecular dynamic simulations they estimated the average phononic thermal energy exchange between the nanoparticles and heat source wall, during the short duration of the collision. Using the collision frequency per unit area of the heat source and the average thermal energy pickup by the nanoparticle, the enhancement of thermal conductivity in nanofluids has been estimated.

One of the important limitations of the model by Ghosh et al. [16] is that it considers only phononic heat exchange between the nanoparticle and the wall during the collision. Karthik et al. [23] extended the model by considering both electronic and phononic heat exchange during the collision. In order to do so Karthik et al. [23] used a continuum approach rather than classical molecular dynamics (CMD) approach to estimate the energy exchange between the nanoparticles and wall, because in the CMD approach the movement of electrons cannot be considered. The continuum modelling to estimate the heat exchange between nanoparticles and heat-source wall will be hereafter referred to as meso-continuum modelling.

In the next section a stochastic model for estimating the heat transfer from the heat-source wall to the nanoparticles via the collision-mediated heat transfer mechanism is elaborated. In the present paper the phase-space evolution in the stochastic model developed by Karthik et al. [23] has been modified as follows. The movement of nanoparticles in the fluid has been described approximately by Maxwell–Boltzmann statistics as these nanoparticles will move like large molecules within smaller molecules.

### Meso-continuum model to estimate heat pickup by nanoparticles from the heat source

**Figure 2:**
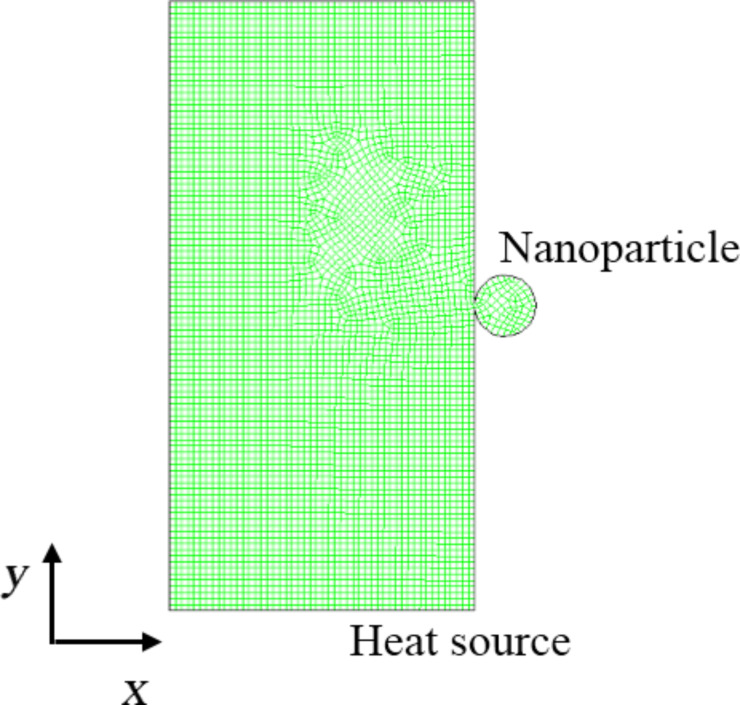
Computational domain of Al_2_O_3_ nanoparticle and heat source used for meso-continuum simulation.

To predict the heat pickup of the nanoparticle during its collision with the heat source a 2D meso-continuum model has been developed. The software package FLUENT 6.3.26 has been used to carry out the simulations. The computational domain used for the simulation is shown in Figure 2.

In Figure 2, the circular section represents the nanoparticle, whereas the rectangular area has been taken as the heat source. Impact dynamics has been used to measure the contact area of the nanoparticle with the heat source [27]. The non-dimensional heat conduction equation is

[1]
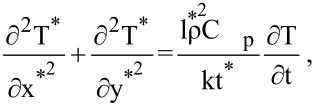


where *C*_p_ is the specific heat capacity, *k* is the thermal conductivity, ρ is the density, *t* is time, and *T* is the temperature.

Initially, the temperature of the heat source is 370 K, and the temperature of the nanoparticle has been set to different temperatures, namely 300 K, 320 K, 333 K and 353 K. The material properties used in the meso-continuum simulations are shown in Table 1.

**Table 1:** The properties of Al_2_O_3_ used in meso-continuum simulation [28].

physical properties	value

bulk thermal conductivity (W/m·K)	35
density (g/cm^3^)	3.96
specific heat capacity (J/kg·K)	880

The heat exchange between the heat source and the particle takes place only during the collision period (∆*t*), which can be determined from the impact dynamics [27] as

[2]
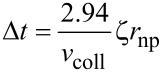


with

[3]
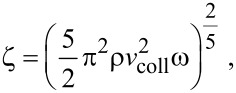


where *r*_np_ is the radius of nanoparticle (Figure 2), *v*_coll_ is the nanoparticle velocity, ρ is the nanoparticle density, ω is the elastic parameter for the nanoparticle. The elastic parameter ω is defined by

[4]
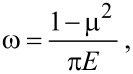


where *E* is the elastic modulus and µ is the Poisson’s ratio. By averaging the temperature of all the grid points within the nanoparticle area in Figure 2, the temperature of the nanoparticle after the collision can be estimated.

### Stochastic model of heat transfer between heat source wall and fluid via nanoparticles

The stochastic model recognizes that nanoparticles in a fluid undergo Brownian movement and frequently collide with the heat source. The recurrence of collision depends on parameters of Brownian motion such as the temperature of the fluid, the size of the nanoparticles and the viscosity of the fluid. Depending on the temperature difference, exchange of heat from the moving nanoparticle to the encompassing fluid occurs via convection, while during the collision of a nanoparticle with the heat source, heat will be transferred via conduction. The phase-space evolution of nanoparticles has been estimated using Maxwell–Boltzmann statistics as

[5]
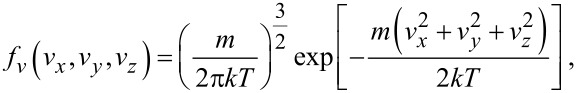


where *f_v_*(*v_x_*,*v_y_*,*v_z_*) is the probability-density function for the velocity of the nanoparticle, *m* is the mass of the nanoparticle, *k* is the Boltzmann constant, and *T* is the temperature of the nanoparticle. Based on the position and velocity of the nanoparticle, the temperature variation of nanoparticle with time, has been calculated by considering the following:

1. During Brownian motion, heat is exchanged when the nanoparticle comes in contact with the heat source.
2. Exchange of heat between the nanoparticles by collision has not been considered, because of the small volume fraction of nanoparticles in the nanofluid.
3. After the collision, heat transfer due to convection takes place between the nanoparticle and the surrounding fluid when the particle moves randomly in the course of its Brownian motion.

Using the Reynolds number and the Prandtl number, the Nusselt number for the flow past a spherical nanoparticle can be estimated using the relation

[6]
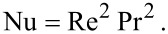


The heat transfer coefficient can be calculated using the Nusselt number as

[7]
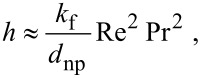


where, *k*_f_ is the thermal conductivity of the fluid and *d*_np_ is the diameter of the nanoparticle. The variation of the temperature of a nanoparticle with time during its Brownian motion can be estimated using

[8]
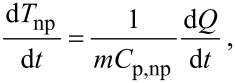


where *T*_np_ is the nanoparticle temperature, *Q* is the heat transfer between the nanoparticles and the fluid, *t* is the time, *C*_p,np_ is the specific heat of nanoparticle. The rate of heat transfer between the fluid and the nanoparticle is measured using

[9]
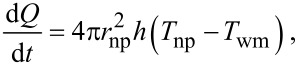


where *T*_wm_ is the temperature of water medium, which depends on the position. The thermal boundary layer in the base fluid has been considered to include the variation in *T*_wm_. The thermal boundary layer thickness is estimated by the relation

[10]
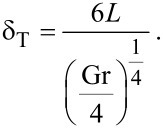


Here *L* is the length of the heat source and Gr is the Grashof number:

[11]
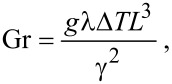


where λ is the volume expansion coefficient of water, ∆*T* is the difference in temperature between the fluid and the nanoparticle, and *g* is the gravitational acceleration.

According to the law of impact dynamics, the particle bounces back when it hits the heat source. By generating a linear expression from meso-continuum simulation, the heat pickup of the nanoparticle has been estimated. In the stochastic simulation, the time step taken is 10^−5^ s [16]. For measuring the enhancement of thermal conductivity of the nanofluid, the collisions taking place in a time frame of 1 s have been considered.

The enhanced thermal conductivity of the nanofluid based on the model of collision-mediated heat transfer can be measured by the ratio of the heat transfer due to collision alone to the convective heat transfer through the fluid without particles. The heat transferred due to collision (*q*_coll_) has been measured as [16]

**Figure 3:**
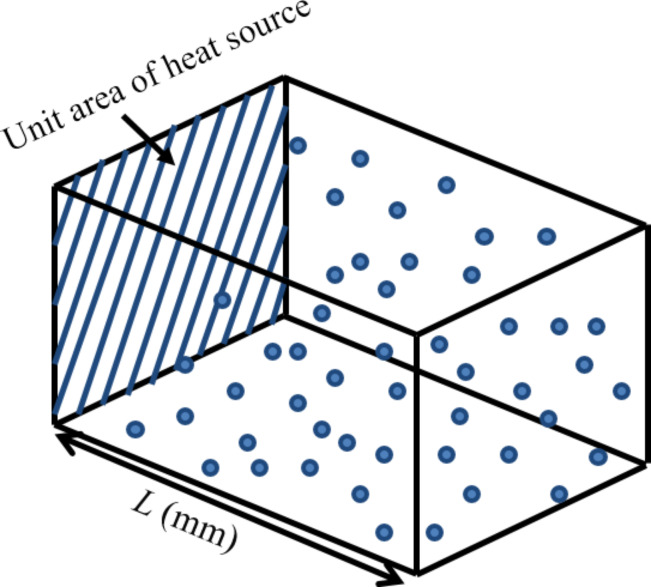
Collision mediated heat exchange mechanism.

[12]
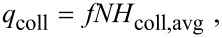


where *f* is the average frequency of collisions of a single nanoparticle per unit area of heat source, *N* is the number of the nanoparticles within the modelled volume, one face of which is the unit area of the heat source (Figure 3), and *H*_coll,avg_ is the average heat transfer per collision. The value of *f* has been estimated using a stochastic model [16,23] and found to vary with the length *L* of the volume for a given volume fraction *V*_f_ of the nanoparticles. Heat transfer per collision can be estimated using the relation

[13]
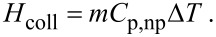


The heat transfer through the thermal boundary layer per unit area per unit time has been expressed by Ghosh et al. [16] and Karthik et al. [23] as

[14]
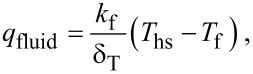


where *T*_hs_ is the heat source temperature, *T*_f_ is the fluid temperature. The percentage enhancement of thermal conductivity of the nanofluid due to the presence of nanoparticles has been calculated using [16] the relation

[15]
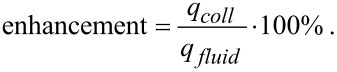


## Measurement Principles of LFM and THWM

**Figure 4:**
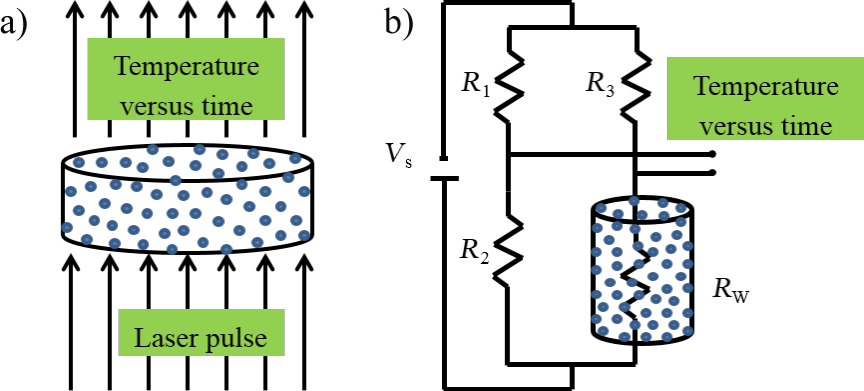
Schematic representation (not to scale) of the experimental method for measuring the thermal conductivity of nanofluids by means of (a) laser flash equipment and (b) transient hot-wire method.

The NETZSCH LFA 447 NANOFLASH equipment measures the thermal diffusivity, specific heat and thermal conductivity using the flash diffusivity method. The liquid is placed into a cylindrical sample holder, whose diameter and depth are 12.7 mm and 0.310 mm, respectively, as illustrated in Figure 4a. The front surface of the specimen holder is then exposed to a finite amount of radiant energy using the laser. Due to the laser pulse, the heat is transported through the sample, which causes a rise in temperature at the rear surface of the sample. This temperature increase as a function of time is recorded with the help of an infrared detector. The thermal diffusivity or thermal conductivity of the sample is computed by taking into account the time taken to obtain half the maximum rise in temperature on the rear face and the specimen thickness.

The transient hot-wire method (THWM) has been widely used to measure the thermal conductivity of nanofluids. The LAMBDA equipment (Flucon fluid control GmbH) is typically employed for transient hot-wire measurements. The sample holder is 90 mm long and 35 mm wide. A 45 mm long and 100 µm diameter metallic wire, which acts both as a source of heat and temperature sensor, is submerged into the liquid sample (Figure 4b). While the wire is electrically heated, the change in resistance of the wire, thus its temperature, is measured as a function of time using a Wheatstone bridge circuit and data acquisition system. The thermal conductivity can be derived directly from the resulting change in the temperature over a known time interval.

### Differences in measured values obtained by LFM and THWM

**Figure 5:**
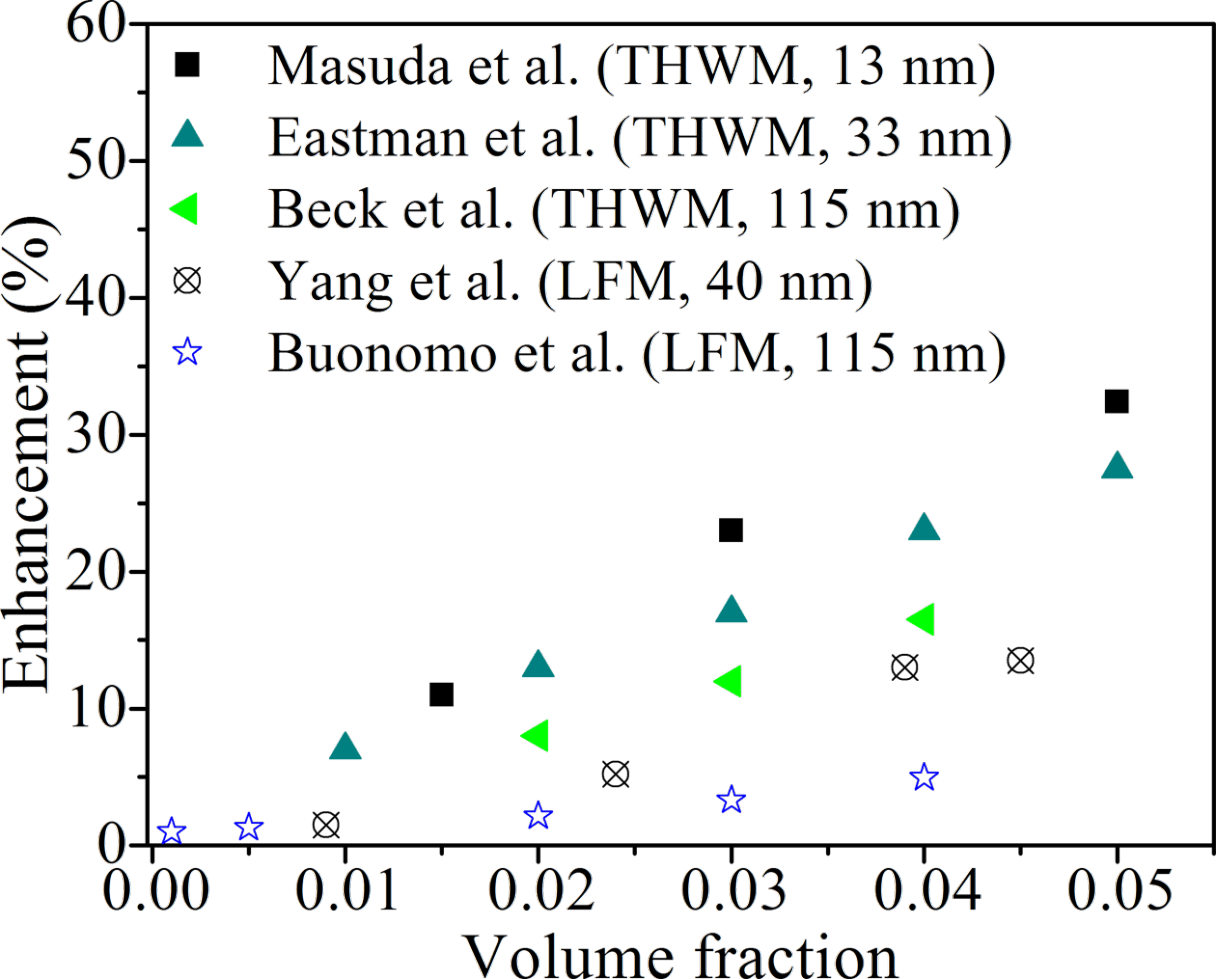
Enhancement in thermal conductivity of water-based Al_2_O_3_ nanofluids measured by the transient hot-wire method (THWM) and the laser flash method (LFM) for different average sizes of Al_2_O_3_ nanoparticles.

A close look into the literature shows that thermal conductivity and its enhancement in the nanofluids obtained by LFM are found to be significantly lower than those measured by THWM. Figure 5 shows the thermal conductivity enhancement of water-based Al_2_O_3_ nanofluids obtained experimentally by Masuda et al. [29], Yang et al. [30], Eastman et al. [31], Buonomo et al. [24] and Beck et al. [4] by THWM or LFM as indicated.

It is evident that the enhancement in the thermal conductivity increased with the increase in the volume fraction of dispersed nanoparticles, and diminished with the increase in the particle size. However, the thermal conductivity enhancement obtained by LFM is always much lower than that obtained by THWM for similar volume fractions of Al_2_O_3_. This observation is anomalous, because this kind of difference in thermal conductivity values is not evident in the case of a fluid that does not contain suspended nanoparticles.

## Results and Discussion

Using the model of collision-mediated heat transfer, the enhancement in the thermal conductivity of water-based Al_2_O_3_ nanofluids in typical THWM and LFM measurements has been computed. In the case of LFM measurements, the nanoparticles are confined to a small liquid pool having a depth of 0.3 mm (Figure 4a), which would significantly restrain the Brownian motion of nanoparticles. On the other hand, the Brownian movement of nanoparticles occurs over a dimension of about 40 mm in the case of THWM (Figure 4b). The collision-mediated-transfer model predicts significantly different frequencies of collision of the nanoparticles with heat source for different depths of the liquid layer. It will now be demonstrated that the experimentally observed differences between the conductivity values obtained by LFM and THWM can be quantitatively accounted for on the basis of the present collision-mediated heat transfer model of nanofluids.

**Figure 6:**
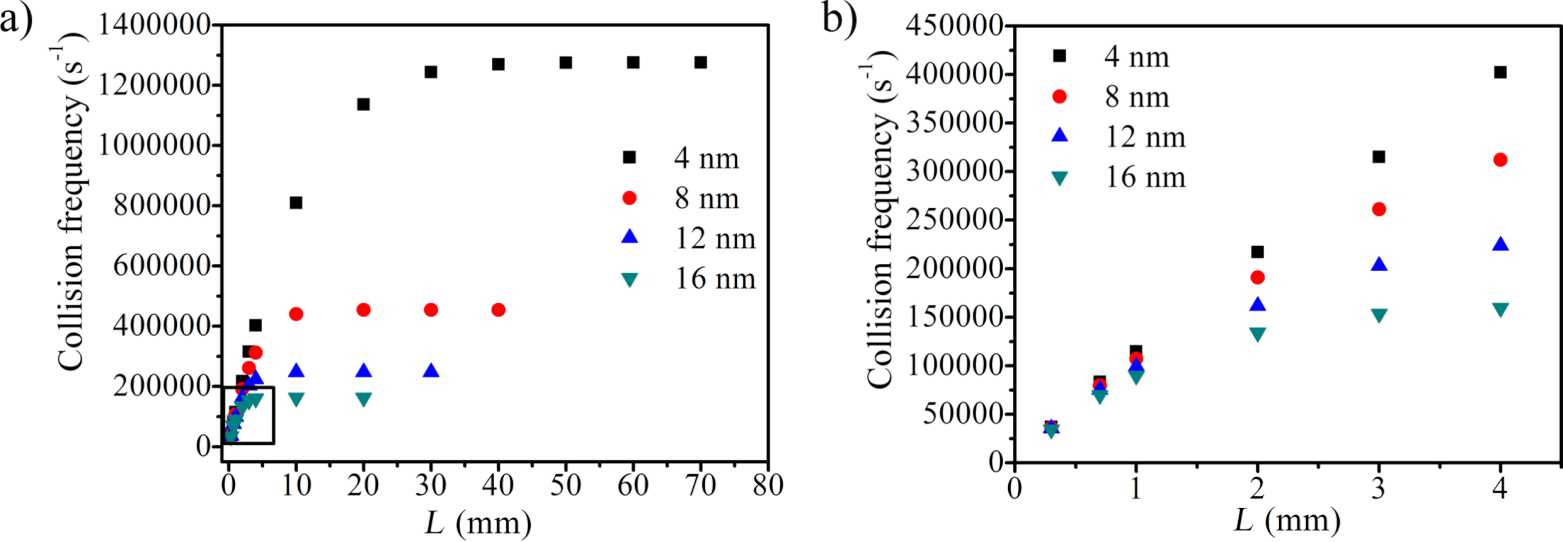
(a) Simulated collision frequency for different size of nanoparticles with *L*, the available distance for Brownian motion. (b) A magnified view of the region within the square in panel a.

**Figure 7:**
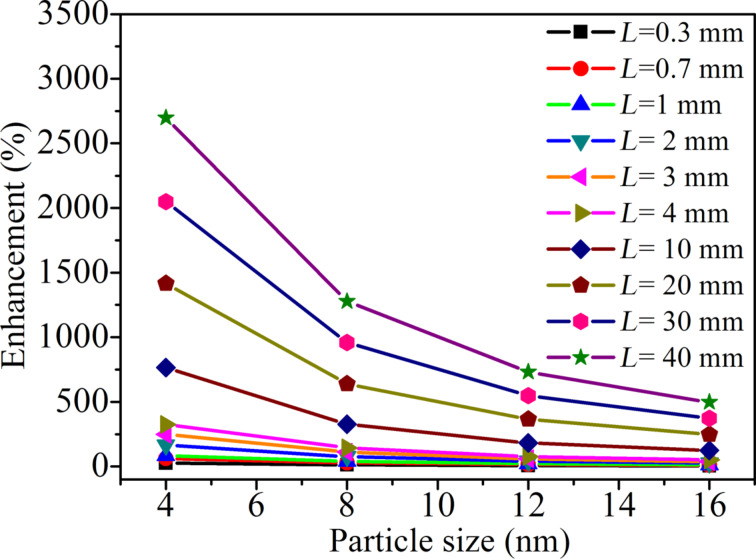
Enhancement of the thermal conductivity predicted for various widths *L* of the nanofluid as a function of the Al_2_O_3_ particle size for a volume fraction *V*_f_ = 0.01.

Figure 6 shows the collision frequency as a function of L for different sizes of nanoparticles. The frequency of collision of nanoparticles per unit area of heat source is a function of *L*, when *L* is smaller than *L*_C_. *L*_C_ depends on the size of the nanoparticles. It is evident that the enhancement in the thermal conductivity (Equation 12) strongly depends on *L* (since frequency of collision depends on *L*) when L < *L*_C_. The predicted enhancement in the thermal conductivity versus *L* for *V*_f_ = 0.01 is shown in Figure 7. It can be seen from the figure that with increase in the particle size or with decrease in the *L* the enhancement in the conductivity decreases.

**Figure 8:**
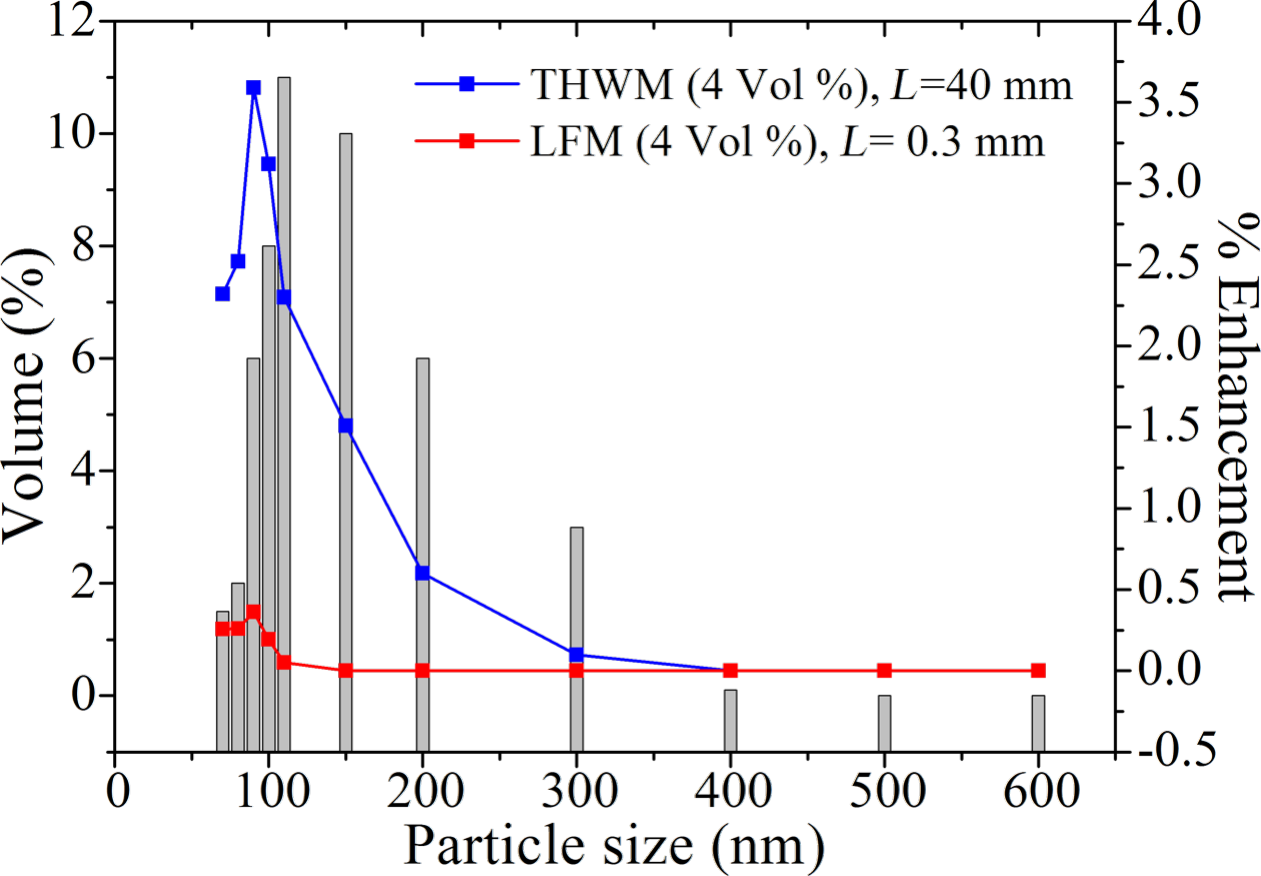
Al_2_O_3_ particle size distribution in nanofluids and its relative contribution to the thermal conductivity enhancement (*V*_f_ = 0.04) by the model presented here for typical values of *L* in THWM (40 mm) and LFM (0.3 mm).

However, it is difficult to obtain particles of only one size during the synthesis process. In reality, nanofluids will always contain a mixture of different sizes of nanoparticles. Let us now theoretically estimate the enhancements in the thermal conductivity of Al_2_O_3_ nanoparticle dispersed aqueous nanofluid in both the situations, i.e., confined in the sample holder of a typical LFM equipment and the fluid surrounding the platinum hot wire of the THWM setup, using the model of collision-mediated heat transfer. To calculate the thermal conductivity of real nanofluids using collision-mediated heat transfer we consider the particle size distributions of Buonomo et al. [24] and Beck et al. [4] for *V*_f_ = 0.04 in both THWM and LFM. Figure 8 shows volume percentage of the particles and their relative contribution to the total enhancement. The summation of the relative thermal conductivity enhancements gives the total enhancement in the thermal conductivity of synthesized Al_2_O_3_ nanofluids having the particle size distributions shown in Figure 8.

**Figure 9:**
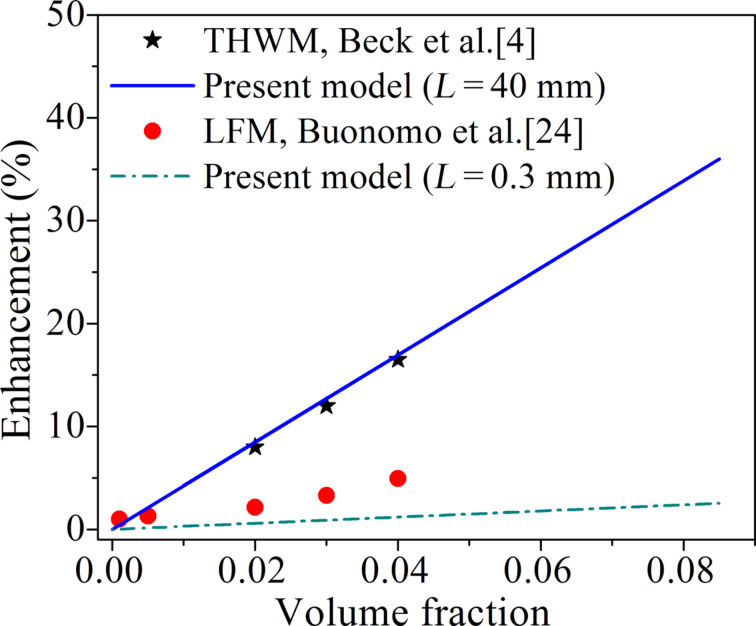
Thermal conductivity enhancement of water-based nanofluids containing Al_2_O_3_ particles of an average size of 115 nm measured experimentally (discrete points) by transient hot-wire method (Beck et al. [4]) and laser flash method (Buonomo et al. [24]) as a function of the nanoparticle volume fraction, compared with the conductivity predicted (lines) by the present model for typical values of *L* in THWM (40 mm) and LFM (0.3 mm).

Figure 9 shows both computed as well as experimentally measured enhancement in thermal conductivity as a function of the volume fraction for both THWM and LFM. It is apparent from Figure 9 that if *L* = 0.3 mm (typical for an LFM measurement), the collision frequency and thermal conductivity enhancement of a nanofluid (*V*_f_ = 0.065 and particle size of 115 nm) predicted by the collision mediated model are 1.1 × 10^16^ s^−1^ and 1.9%, respectively. The predicted collision frequency and thermal conductivity enhancement for the same nanofluid are 1.6 × 10^17^ s^−1^ and 27.5%, if *L* ≥ 40 mm (typical for THWM). The thermal conductivity enhancement predicted by the collision-mediated heat transfer mechanism was found to be in good agreement with the literature for *L* = 40 mm (THWM), whereas it is slightly underestimating when *L* = 0.3 mm (LFM).

Thus, the big difference in the thermal conductivity enhancement measured by the LFM and the THWM can be attributed to the significant difference in the constraints on the Brownian motion of nanoparticles in nanofluids affecting the frequency of collision with the heat source. This also points at the potential limitation of using nanofluids in micro-channels for enhanced heat transfer applications, because of the limited space available there for the Brownian movement of nanoparticles in two dimensions as compared with *L*_C_, which would make collision-mediated heat transfer by nanoparticles mostly ineffective.

## Conclusion

The collision-mediated heat transfer models for nanofluids proposed earlier by Ghosh et al. [16] and Karthik et al. [23] have been further modified to show that a small volume (about 50 µL) normally used in the laser flash method (LFM) severely restricts the Brownian motion of particles compared to the much larger volume (more than 50 mL) available in the transient hot-wire method (THWM). As a result the enhancement of the thermal conductivity of any given nanofluid measured by LFM is predicted to be about one order of magnitude lower than that obtained by THWM, a fact shown by experiments with aqueous Al_2_O_3_ nanofluids reported by other investigators. This analysis also gives an indication that nanofluids are unlikely to be a more effective coolant in micro-channels.
